# Understanding the Roles of the NSD Protein Methyltransferases in Head and Neck Squamous Cell Carcinoma

**DOI:** 10.3390/genes13112013

**Published:** 2022-11-02

**Authors:** Madhavi Murali, Vassiliki Saloura

**Affiliations:** 1 Thoracic and GI Malignancies Branch, Center for Cancer Research, National Cancer Institute, Bethesda, MD 20892, USA; 2 School of Medicine, The University of Missouri-Kansas City, Kansas City, MO 64018, USA

**Keywords:** NSD, protein methyltransferases, HPV-positive, HPV-negative, head and neck squamous cell carcinoma, HNSCC

## Abstract

Head and neck squamous cell carcinoma (HNSCC) is the sixth most prevalent non-skin cancer in the world. While immunotherapy has revolutionized the standard of care treatment in patients with recurrent/metastatic HNSCC, more than 70% of patients do not respond to this treatment, making the identification of novel therapeutic targets urgent. Recently, research endeavors have focused on how epigenetic modifications may affect tumor initiation and progression of HNSCC. The nuclear receptor binding SET domain (NSD) family of protein methyltransferases NSD1-NSD3 is of particular interest for HNSCC, with NSD1 and NSD3 being amongst the most commonly mutated or amplified genes respectively in HNSCC. Preclinical studies have identified both oncogenic and tumor-suppressing properties across NSD1, NSD2, and NSD3 within the context of HNSCC. The purpose of this review is to provide a better understanding of the contribution of the NSD family of protein methyltransferases to the pathogenesis of HNSCC, underscoring their promise as novel therapeutic targets in this devastating disease.

## 1. Introduction

Head and neck squamous cell carcinoma (HNSCC) is the sixth most prevalent non-skin cancer in the world and is often found to have a poor prognosis [1,2,3]. HNSCC can be categorized into two types: human papilloma virus-positive (HPV-positive), which is associated with viral exposure to HPV, and human papilloma virus-negative (HPV-negative), which has strong associations with alcohol and tobacco use. Given the poor treatment outcomes particularly in HPV-negative HNSCC, understanding the molecular basis for how these types of cancers develop is of paramount importance for the identification of novel therapeutic approaches. 

HNSCC, particularly HPV-negative tumors, demonstrate a plethora of genetic and expression alterations in protein methyltransferases and demethylases [4,5,6], with a significant body of preclinical evidence supporting the importance of this class of enzymes in the pathogenesis of this disease [7]. Protein methyltransferases and demethylases comprise a family of enzymes that, respectively, write or erase methyl groups on lysine or arginine residues of histone and/or non-histone substrates. Of particular interest for HNSCC are the nuclear receptor binding SET domain (NSD) 1 (NSD1), NSD2, and NSD3 protein lysine methyltransferases that mono- and di-methylate lysine 36 of histone H3 (H3K36) [8,9,10,11,12,13,14,15]. Methylated H3K36 characterizes actively transcribed euchromatin. *NSD1* and *NSD3* are amongst the top ten most frequently genetically altered genes in HPV-negative HNSCC, with *NSD1* carrying mutations and *NSD3* coamplifying with fibroblast growth factor receptor 1 (FGFR1) (8p11-12 amplicon) in HPV-negative HNSCC [16]. 

The NSD family of protein lysine methyltransferases play a critical role in regulating gene expression, oncogenesis, tumor suppression, and cell differentiation, with mutations, amplifications, or translocations of these genes associated with cancer development and progression in multiple cancer types [17,18]. *NSD1* (*KMT3B*) was initially discovered as a driver for de novo pediatric acute myeloid leukemia (AML) with the recurrent t(5;11)(q35;p15.5) translocation [19]. It was found as a fusion partner of the nucleoporin gene (*NUP98*) transcript, constituting the chimeric messenger RNA NUP98-NSD1, the most frequent fusion reported in pediatric AML [20]. Wang et al. [21] subsequently showed that the NUP98-NSD1 fusion protein induces AML in vivo, enforces myeloid cell stemness, and upregulates the expression of *HoxA7* and *HoxA9* by binding to adjacent genomic elements, maintaining H3K36 methylation and histone acetylation, and antagonizing EZH2-mediated repression of the *Hox-A* locus genes. Inactivating mutations of *NSD1* prevented *Hox-A* gene activation and myeloid progenitor immortalization, further supporting the role of NSD1 in pediatric AML. 

*NSD2* (*WHSC1* or *MMSET*) was first discovered as part of the 4p16.3 chromosomal region which is deleted in Wolf–Hirschhorn syndrome [22], as well as a component of the t(4;14)(p16.3;q32.3) translocation, placing it under the regulatory genomic regions of the immunoglobulin locus [22,23]. This translocation is found in approximately 20% of patients with multiple myeloma and is associated with *NSD2* and *FGFR3* (co-amplified) overexpression [24], though *NSD2* is purported to exert the primary oncogenic role. The oncogenic function of NSD2 has been primarily studied in multiple myeloma with the t(4;14) translocation. Knockdown of NSD2 in these cell lines decreases proliferation, cell cycle progression, and DNA repair and increases apoptosis and adhesion [25,26,27,28]. NSD2 induces these phenotypes through an increase and redistribution of H3K36 dimethylation which enhances gene expression. Concurrently, *NSD2* overexpression is associated with a global reduction in the repressive H3K27me3 mark, which is deposited in specific chromatin regions, leading to inappropriate silencing of genes [29,30]. 

*NSD3* was first discovered by two independent groups in 2001 [31,32]. Angrand et al. reported that *NSD3* was mapped to the 8p12 chromosomal band which was found amplified in breast cancer cell lines [31]. Stec et al. [32] reported *NSD3* to be mapped at 8p11.2, a region that is frequently rearranged in many human cancer types. Interestingly, *NSD3* was coamplified with *FGFR1*, similarly to *NSD2* being coamplified with *FGFR3*. *NSD3* is amplified in multiple cancer types, such as bladder, breast, lung, liver, and colorectal cancer, as well as head and neck cancer [33]. NSD3 knockdown decreased proliferation in 8p11-12 amplified breast cancer cell lines [34], as well as cell cycling in breast, bladder, and lung cancer cell lines [35,36], implicating NSD3 in cell cycle regulation. NSD3 has also been identified as an oncogenic driver of lung squamous cell carcinoma [37]. Yuan et al. established NSD3, and not FGFR1, as a principal oncogenic driver in lung squamous cell carcinoma. Ablation of NSD3, but not FGFR1, attenuated tumor growth in a mouse model of lung squamous cell carcinoma. Accordingly, NSD3 depletion in patient-derived xenografts from primary lung squamous cell carcinoma samples with *NSD3* amplification attenuated tumor growth. This work establishes *NSD3* as a primary oncogenic driver in lung squamous cell carcinomas with the 8p11-12 amplification. 

The goal of this review is to summarize the literature available on the functions of the NSDs in HNSCC and highlight their importance as oncogenic drivers and thus as potential novel therapeutic targets in this disease. For a more comprehensive review of the oncogenic functions of the NSDs in all cancer types, we recommend an excellent review by Bennett et al. [17].

## 2. Materials and Methods

We utilized the PubMed database to systematically interrogate and identify original research articles and literature reviews that investigate the NSD protein family and protein structure, NSD tissue expression profiles, prognostic implications, and mechanisms of NSD in HNSCC, and currently available NSD inhibitors. We used the search terms “NSD family”, “NSD1 and squamous cell cancer”, “NSD2 and squamous cell cancer”, “NSD3 and squamous cell cancer”, “NSD1 and head and neck cancer”, “NSD2 and head and neck cancer”, and “NSD3 and head and neck cancer”, to identify the above discussion areas. We included studies published only in peer-reviewed journals. Additionally, we narrowed the scope to include articles that investigated clinicopathologic and/or mechanistic implications of NSD in the context of HNSCC. 

## 3. Results and Discussion

### 3.1. Structure of NSD Family of Protein Methyltransferases

Overall, the NSD family of protein lysine methyltransferases have multiple common domains across NSD1, NSD2, and NSD3. All three have a catalytic suppressor of variegation 3–9, Enhancer of zeste and Trithorax (SET) domain, a high-mobility-group box, two proline-tryptophan-tryptophan-proline (PWWP) domains, and five plant homeodomain zinc fingers. The SET domain within the NSD protein directs the methyltransferase activity of the NSDs, the PWWP domains mediate binding to methylated histone H3 and DNA, and plant homeodomain-type zinc finger domains are involved in interactions with chromatin in addition to other proteins [38,39,40,41,42,43]. 

NSD1 is the largest of the NSD family members, consisting of 2696 amino acids. Its gene is located on chromosome 5q35 [41]. It has two nuclear receptor interaction domains and binds to androgen, estrogen, and retinoic acid receptors [44]. NSD2 has the smallest molecular weight of the proteins in the NSD family, consisting of 1365 amino acids. It has three protein isoforms of differing amino-acid lengths, determined by alternative splicing: the NSD2 type I, NSD2 type II, and the interleukin-5 response element II binding protein (RE-IIBP). NSD2 type II and RE-IIBP share a SET domain as well as a region of 584 amino acids within the carboxy-terminal end. The NSD2 type I isoform does not have a SET domain and has no protein methyltransferase activity [41]. NSD3 is the least characterized of the NSD family of protein methyltransferases. It is located on chromosome 8p12 and has two main isoforms that share a common N-terminal part. NSD3L (“long”) has 1437 amino acids, and NSD3S (“short”) has 645 amino acids. Similar to the NSD2 type I isoform, the NSD3S isoform lacks a SET domain [41].

### 3.2. Substrates of NSD Protein Methyltransferases

NSD1, NSD2, and NSD3 mono- and di-methylate lysine 36 on histone H3. SETD2 then methylates the dimethylated H3K36 (H3K36me2) to generate trimethylated H3K36 (H3K36me3). These histone marks activate gene expression within euchromatin regions [45,46]. Specific DNA and histone-specific points between the nucleosome target and NSD2 or NSD3 allow for methylation specificity of targets such as H3K36 [47]. NSD2 has also been found to directly monomethylate histone H1 at lysine 85, leading to stem cell-like characteristics in HNSCC [48].

Non-histone substrates of the NSDs have also been reported. NSD1 directly methylates K218 and K221 of the RELA/p65 subunit of nuclear factor kappa-light-chain-enhancer of activated B cells (NF-κB), inducing activation of NF-κB, while demethylation of NF-κB by FBXL11 inactivates it. Interestingly, NF-κB activates the expression of FBXL11, indicating the presence of a negative feedback loop modulated by the reversible lysine methylation of this key transcription factor [49]. NSD2 dimethylates phosphatase and tensin homolog (PTEN) at K349, which is in turn recognized by 53BP1 to recruit PTEN on DNA double-strand breaks and enable DNA repair in colon cancer cells [50]. NSD3 interacts with and methylates interferon regulatory factor 3 (IRF3) and the epidermal growth factor receptor (EGFR) [51,52]. Specifically, NSD3 binds to IRF3 through its PWWP1 domain and methylates IRF3 at K366. This methylation promotes the dissociation of protein phosphatase PP1cc from IRF3, enhancing its phosphorylation and thus its transcriptional activity, and ultimately promoting type I interferon production and antiviral innate immunity [51]. Additionally, NSD3 directly methylates EGFR at K721, with oncogenic effects described in more detail in Section 3.4 [52] [Table 1]. 

### 3.3. Physiological Functions of NSD1, NSD2, and NSD3

NSD1 and NSD2 knockout in mice is lethal, thus suggesting indispensable roles of NSD1 and NSD2 in normal tissues, as well as non-redundant functions of NSD1, NSD2, and NSD3 [11,53]. Inactivating germline mutations in NSD1 are associated with the Sotos syndrome, a childhood developmental delay syndrome that is characterized by craniofacial abnormalities, advanced bone age, variable learning disabilities, and an increased risk of developing malignancies [54,55]. NSD1 mutations are also associated with the Beckwith–Wiedemann syndrome, characterized by exophthalmos, macroglossia, visceromegaly, and a higher risk of embryonal tumors [55]. Germline NSD2 deletions are associated with the Wolf–Hirschhorn syndrome, which is characterized by heart defects, developmental delays, epilepsy, and craniofacial and midline fusion abnormalities [22,56]. The physiological function of NSD3 has yet to be fully elucidated, as no relevant overgrowth syndromes attributed to defects in this gene have been described in humans.

### 3.4. Pathobiological Implications of the NSDs in HNSCC

Given the significant differences in the pathogenesis of HPV-negative versus HPV-positive HNSCC, this section is commenting on the reported functions of the NSDs in these cancer types separately, wherever feasible. 

#### 3.4.1. The NSDs in HPV-Negative HNSCC

**Figure 1:**
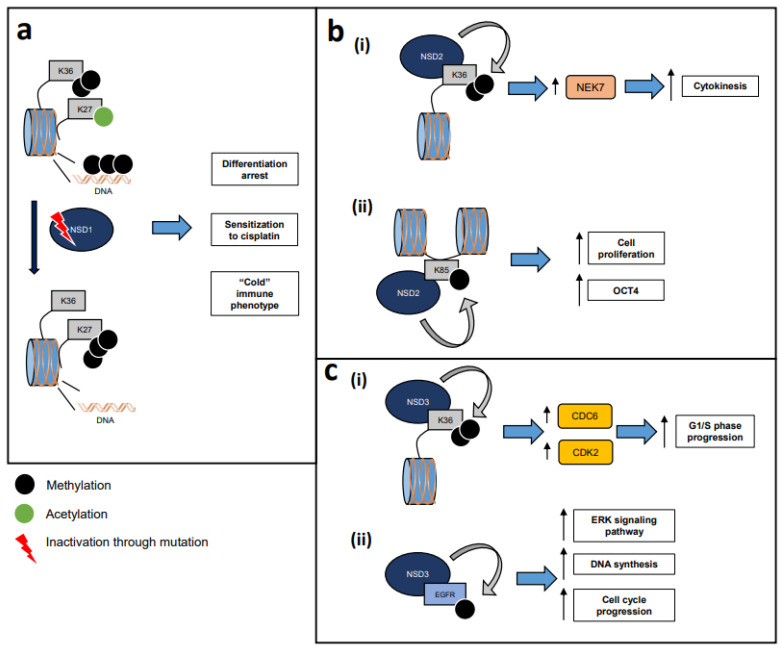
Reported mechanisms of action of the NSDs in HPV-negative HNSCC. (**a**) Inactivating mutations of NSD1 in HPV-negative HNSCC induce decreased levels of H3K36me2, global DNA hypomethylation and increased levels of H3K27me3, leading to differentiation arrest, increased sensitivity to cisplatin, and a “cold” immune phenotype. (**b**) NSD2 in HNSCC. (i) NSD2 dimethylates H3K36, leading to NIMA-related kinase 7 (NEK7) upregulation to promote cytokinesis. (ii) NSD2 monomethylates H1K85, promoting pathways related to cell proliferation and upregulating OCT4, a stemness factor. (**c**) NSD3 in HNSCC. (i) NSD3 dimethylates H3K36, leading to CDC6 and CDK2 upregulation and promotion of G1-S phase progression. (ii) NSD3 methylates epidermal growth factor receptor (EGFR) at lysine 721, increasing interaction with PCNA to enhance DNA synthesis and promote cell cycle progression.

The Cancer Genome Atlas (TCGA) has shown that NSD1 and NSD3 are among the most frequently genetically altered genes in HPV-negative HNSCC, with NSD1 carrying splice-site, nonsense, or frame-shift mutations, and NSD3 coamplifying with FGFR1 (8p11-12 amplicon) in HPV-negative HNSCC [16], supporting their importance in the pathogenesis of this disease [Table 2, Figure 1]. 

#### 3.4.2. NSD1 in HPV-Negative HNSCC

Pertaining to NSD1, Papillon-Cavanagh et al. first reported a subset of HPV-negative HNSCC tumors of the TCGA comprising 13% of all tumors, characterized by recurrent p.Lys36Met (K36M) mutations whereby lysine 36 is replaced by methionine, as well as predominantly non-overlapping NSD1 inactivating mutations [57]. A study by Pan et al. [58] corroborated these findings with 13% of HPV-negative HNSCC tumors containing genetic alterations in *NSD1*, with the majority constituting truncating mutations (61.3%), missense point mutations (30.7%), homozygous deletions (6.7%) and in-frame deletions (1.3%). The specific cluster described by Papillon-Cavanagh et al. was also characterized by strong global DNA hypomethylation, decreased H3K36me2 but intact H3K36me3 levels, suggesting that defects in the H3K36 methylation pathway may also potentially drive DNA hypomethylation. This DNA hypomethylated cluster was also enriched in TP53 and CASP8 mutations and a heavy smoking history, and comprised of the oral cavity and larynx tumors. These genomic findings were validated in an independent cohort of HPV-negative HNSCC tumor samples with immunohistochemistry showing that the K36M mutant histone was exclusively expressed in HNSCC cells and was associated with decreased global levels of H3K36me2 and H3K36me3, and NSD1 loss was associated with decreased H3K36me2. Transcriptomic analysis of this H3K36M/NSD1 mutant subset revealed enrichment in cellular differentiation pathways, supporting a pathogenetic model whereby these mutations impair H3K36 methylation and lead to a differentiation arrest of keratinocytes.

In a study reported approximately at the same time as the Papillon-Cavanagh study, Peri et al. [59] reported for the first time the prognostic significance of *NSD1* or *NSD2* mutations in patients with HPV-negative laryngeal HNSCC. Particularly, patients with mutations in either *NSD1* or *NSD2* had a better clinical outcome compared to patients with wild-type *NSD1* or *NSD2*. In a subsequent study, *NSD1*-mutant HPV-negative HNSCC patients were also found to have a significant overall survival advantage over *NSD1*-wild-type patients [60]. *NSD1* CRISPR knockout HPV-negative HNSCC cell lines demonstrated CpG DNA hypomethylation and a significant increase in cisplatin sensitivity, implying that *NSD1*-mutant, DNA hypomethylated HPV-negative HNSCC patients may have a favorable chemotherapeutic response to cisplatin. Similar findings were reported by Pan et al. [58], who showed that NSD1 depletion in HPV-negative HNSCC cell lines increased their sensitivity to cisplatin and carboplatin.

To comprehensively investigate the impact of NSD1 mutations on the genome-wide distribution of histone marks and on the transcriptional profile of HPV-negative HNSCC cells, Farhangdoost et al. [61] conducted genome-wide chromatin immunoprecipitation and whole-genome bisulfite sequencing combined with quantitative mass spectrometry in NSD1-wild-type versus NSD1-mutant HNSCC cell lines. NSD1 depletion induced the loss of intergenic H3K36me2, global DNA hypomethylation, and gain of H3K27me3 in intergenic regions, reducing the expression of target genes and pathways, including KRAS signaling, EMT, and inflammatory responses [61]. Furthermore, in enhancer regions, *NSD1*-mutant cell lines demonstrated loss of H3K36me2, DNA methylation, and H3K27Ac, while gaining H3K27me3, implying decreased enhancer activity that correlated with reduced expression of target genes [61]. 

Interestingly, the NSD1-mutant DNA hypomethylated HNSCC subtype has been associated with poor CD8+ T-cell infiltration, in contrast to cancers such as melanoma, in which DNA hypomethylation has been implicated in enhanced immune responses through viral mimicry mechanisms and re-expression of endogenous retroviral elements [62]. Brennan et al. injected the flanks of NOD-scid IL2Rgamma^null^ mice with control or NSD1 shRNA knockdown HNSCC cells and once tumors were established, mice were injected with mouse peripheral blood mononuclear cells. Ten days later, flow cytometry of the flank tumors revealed significantly lower infiltration of CD8+ T-cells as well as M1 macrophages in the NSD1 knockdown tumors, implying that NSD1-mutant tumors may be immune evasive and resistant to immunotherapy. Li et al. [63] further interrogated the function of NSD1 in squamous cell carcinomas of the head, neck, and lung, and found that *NSD1* loss through knockout or inactivating mutations induced downregulation of interferon-stimulated genes (ISGs) in HPV-negative HNSCC and lung squamous cell lines, despite DNA hypomethylation and upregulation of LINE-1 and SINE/Alu retransposons. *NSD1*-mutant HNSCC cell lines were also unable to express ISGs in response to pathogen-associated molecular patterns, such as lipopolysaccharide. *Nsd1* conditional knockout mice further demonstrated decreased CD8+ T-cell, macrophage, and NK cell infiltration in carcinogen-induced tongue carcinoma lesions. Interestingly, *NSD1* KO was associated with a genome-wide depletion of H3K36me2 and a concordant increase in H3K27me3 in the promoters of transcriptionally downregulated genes, among which the *IFNA* family genes, such as *IFNLR1* and *IL19*, were enriched. The authors further functionally analyzed *IFNLR1*, which encodes for the receptor of type III IFN lambda, and found its promoter enriched in H3K27me3 in *NSD1* KO cells, and its protein expression levels reduced in *NSD1* KO cells and *Nsd1*-deleted mouse tumors. Both human and mouse HNSCC cell lines with *NSD1* ablation showed impaired interferon signaling responsiveness to IFN-λ. Treatment of NSD1-deficient HPV-negative HNSCC cells with the FDA-approved EZH2 inhibitor tazemetostat restored the expression of IFNLR1 and ISGs, while depletion of IFNLR1 abolished the rescue of ISG expression mediated by EZH2 inhibition, implying that the effect of EZH2 inhibition is at least partially mediated by interferon lambda and the sensing by its receptor and its downstream signaling pathway. Accordingly, treatment of C57BL/6 syngeneic *Nsd1* KO MOC1 tumors with tazemetostat induced immune cell infiltration and inhibited the growth of these tumors. These findings strongly support the role of NSD1 in tumor immune evasion in HPV-negative HNSCC through dampening of type III IFN responsiveness and suggest that EZH2 inhibition may restore immune cell infiltration through derepression of *IFNLR1* and possibly other ISGs.

Despite the immune “cold” phenotype of these tumors, the aforementioned preclinical studies suggest that NSD1-mutant HPV-negative HNSCC patients may be highly responsive to chemoradiotherapy, and thus may represent a subgroup of patients where de-escalation approaches may be considered. Furthermore, EZH2 inhibition may induce immune cell infiltration and render these tumors responsive to immunotherapy.

#### 3.4.3. NSD2 in HPV-Negative HNSCC

Regarding the function of NSD2 on HNSCC, a study by Saloura et al. found that NSD2 and H3K36me2 levels were significantly higher in both HPV-negative and HPV-positive HNSCC compared to normal and dysplastic epithelium [64]. Knockdown of NSD2 induced growth suppression, apoptosis, and a delay in cell-cycle progression. These effects were possibly mediated through NIMA-related kinase-7 (NEK7), which was found to be directly regulated through NSD2-mediated H3K36 di-methylation.

NSD2 has also been reported to monomethylate K85 of histone H1.4. NSD2 overexpressing HPV-negative HNSCC cells stably transfected with a plasmid expressing wild-type H1.4 had a higher capacity to form spheres in low attachment conditions compared to cells transfected with a plasmid expressing mutant H1.4 that cannot be methylated at K85 (H1.4K85A). Accordingly, cells expressing the wild-type H1.4 had higher expression of OCT4 compared to cells expressing H1.4K85A, while FLAG-tagged wild-type H1.4 showed increased occupancy on the *OCT4* gene compared to FLAG-tagged H1.4K85A, implying that methylation of H1.4K85 may enable the transcription of *OCT4* in HPV-negative HNSCC cells. These findings support that NSD2 monomethylates H1.4 at K85 and drives stemness features through transcriptional upregulation of *OCT4* in HPV-negative HNSCC cells [48].

Importantly, NSD2 has been implicated as a mediator of cisplatin resistance in esophageal SCC, which has a similar genetic background to HPV-negative HNSCC [66]. More specifically, NSD2 knockdown in esophageal SCC cell lines induced sensitization to cisplatin both in vitro and in EC109 xenograft tumors, while NSD2 overexpression augmented resistance to cisplatin. This was mediated by the long non-coding RNAs MACC1-AS1 and FOXD2-AS1, which were transcriptionally regulated through NSD2-mediated H3K36me2 deposition on their promoters. Knockdown of MACC1-AS1 abolished the phenotype of NSD2-mediated cisplatin resistance. Furthermore, higher expression levels of MACC1-AS1 were associated with reduced overall survival in esophageal SCC patients and correlated positively with NSD2 expression levels. 

The above preclinical studies involved NSD2 in cell cycle progression, stemness, and possibly cisplatin resistance primarily in HPV-negative HNSCC.

#### 3.4.4. NSD3 in HPV-Negative HNSCC

NSD3 has also been reported to have oncogenic functions through both histone and non-histone methylation substrates in HNSCC. Saloura et al. found that NSD3 and H3K36me2 play a critical role in the G1 to S phase transition in HNSCC cell lines [65]. NSD3 was significantly overexpressed in patients with HNSCC and was associated with poor grades and heavy smoking history. NSD3 knockdown induced growth suppression and significant cell cycle arrest in both HPV-negative and HPV-positive HNSCC cell lines, and was found enriched in the gene body regions of two important cell-cycle regulators, CDC6 and CDK2. The cell cycle arrest phenotype was reversed with the re-expression of exogenous, enzymatically active, but not enzymatically dead NSD3, suggesting that the enzymatic activity of NSD3 is necessary for cell cycle progression in HNSCC cells [65]. 

NSD3 may also exert oncogenic effects through non-histone methylation pathways in HNSCC [52]. NSD3 monomethylates EGFR at lysine K721 within its tyrosine kinase domain and activates the downstream ERK cascade, a process that occurs independently of the presence of the epidermal growth factor. Furthermore, NSD3-mediated monomethylation of nuclear EGFR increases its interaction with PCNA, enhances DNA synthesis, promotes cell cycle progression, and drives resistance to EGFR inhibition in HPV-negative HNSCC cell lines.

Nuclear protein in testis (NUT) midline carcinoma (NMC) is an aggressive epithelioid malignancy that predominantly occurs in the thorax, head, or neck regions and carries a very poor prognosis [67]. NMC tumors are typically driven by BRD4/3-NUT fusion oncoproteins; however, fusions with non-BRD genes have also been described. Particularly, French et al. [68] described the t(8;15)(p12:q15) chromosomal translocation generating the NSD3-NUT fusion oncoprotein which inhibits cellular differentiation and promotes proliferation in NMC cells [68]. The NSD3-NUT oncoprotein binds to BRD4 and NSD3 is necessary for the blockade of cellular differentiation in NMC cells. Bromodomain inhibitors promote differentiation and inhibit the proliferation of NMC cells, suggesting that the NSD3 oncogenic activity in these NSD3-NUT oncoprotein-expressing NMC cells can be hindered through bromodomain inhibition. Interestingly, Yuan et al. [37] recently reported that *NSD3*-amplified lung squamous cell carcinoma patient-derived xenografts were sensitive to bromodomain inhibition, supporting the role of bromodomain inhibitors in NSD3-driven lung squamous cell carcinomas. Given the background genetic similarity of HPV-negative HNSCC and smoking-related lung squamous cell carcinomas, bromodomain inhibition may be a reasonable therapeutic approach to investigate NSD3-driven HPV-negative HNSCC.

#### 3.4.5. The NSDs in HPV-Positive HNSCC

With the exception of a few preclinical studies mentioned above that attempted to examine the function of the NSDs in both HPV-positive and HPV-negative HNSCC cell lines [64,66], the majority of published preclinical studies have focused on the mutational patterns and the impact of the NSDs on the survival of HPV-positive HNSCC patients. Haft et al. examined the mutational landscape of 46 HPV-positive oropharyngeal HNSCC tumors of the TCGA, as well as a separate exome sequencing dataset of another 46 HPV-positive oropharyngeal HNSCC tumors of a cohort from Johns Hopkins [69]. They found that *NSD1* was mutated in 4.5% and 9% of tumors in the TCGA and the John Hopkins cohort, respectively, and it constituted one of the most frequently mutated chromatin modifiers in these tumors. This finding suggests that *NSD1* mutations play a distinct pathogenetic role in a subset of HPV-positive HNSCC tumors. 

Pan et al. further investigated the survival impact of inactivating *NSD1* mutations in HPV-positive HNSCC using the TCGA database, and found that *NSD1*-mutant HPV-positive HNSCC patients had worse survival compared to *NSD1* wild-type patients, in contrast to HPV-negative HNSCC patients [58]. Although these findings were based on the small cohort of HPV-positive HNSCC patients of the TCGA and would need to be validated in a larger cohort, they support opposing functions of NSD1 in HPV-positive versus HPV-negative HNSCC tumors. 

Gameiro et al. investigated the impact of the mRNA expression levels, but not mutations, of NSD1, NSD2, and NSD3 on the overall survival outcomes in HPV-positive HNSCC patients and contrasted these results to HPV-negative HNSCC patients [70]. Interestingly, expression levels of the NSDs did not correlate with survival in HPV-negative HNSCC, but lower levels correlated significantly with worse overall survival in HPV-positive HNSCC patients. While these results should be interpreted cautiously given the low number of HPV-positive HNSCC patients in the TCGA database, it is interesting to note that while *NSD1* inactivating mutations are associated with improved survival outcomes in HPV-negative HNSCC, lower NSD1 expression levels, which would be presumed to phenocopy the loss of NSD1 function through inactivating mutations, render worse survival outcomes in HPV-positive HNSCC. This is in accordance with Pan et al., [58] who showed that *NSD1* mutations are associated with worse survival in HPV-positive HNSCC. These findings underscore the significant biological differences between HPV-positive and HPV-negative HNSCC tumors, but also highlight the notion that chromatin modifiers, such as NSD1, may exert different biological functions depending on the cell type context. 

### 3.5. Clinical/Translational Implications of the NSDs in HNSCC

The distinct genetic alteration patterns of the NSDs and relevant preclinical studies point towards a definite role of the NSDs in the pathogenesis of HNSCC, particularly in specific subsets of HPV-negative HNSCC. HPV-negative patients with inactivating *NSD1* mutations, albeit harboring a “cold” tumor microenvironment, may be more sensitive to chemoradiotherapy compared to *NSD1*-wild-type patients, and may thus benefit from de-escalation treatment strategies. A randomized phase II study of newly diagnosed HPV-negative HNSCC patients eligible for curative intent chemoradiotherapy prospectively stratified by *NSD1*-mutation status will provide a definitive answer to whether *NSD1*-mutations render chemoradiotherapy sensitivity and merits consideration. Furthermore, with the advent of pembrolizumab as a standard of care therapy in the recurrent/metastatic setting and studies implicating *NSD1* mutations and antagonistic H3K27me3 in immune evasion in HPV-negative HNSCC [62,63], *NSD1* mutations merit clinical investigation as a potential biomarker of poor response to immunotherapy in HPV-negative HNSCC patients, while these patients may be sensitized and gain benefit from immunotherapy with EZH2 inhibition. 

Regarding HPV-negative patients with *NSD3* amplifications, the role of bromodomain inhibitors [37,67,68] merits further investigation. Furthermore, these patients may be resistant to EGFR-based therapeutic approaches and may potentially benefit from cell cycle inhibitors [52,66].

While no specific inhibitors targeting NSD1, NSD2, or NSD3 have been discovered, a number of compounds have been reported to have inhibitory effects on the SET domain of the NSDs ([71,72], Table 3). On the other hand, a number of bromodomain inhibitors are already under different phases of clinical development in various cancer types [NCT05327010, NCT04840589, NCT04471974, NCT05071937] and may be considered for NSD3-driven HPV-negative HNSCC ([73,74,75,76,77], Table 3).

## 4. Conclusions

The aforementioned preclinical studies underscore a distinct role of the NSDs, particularly in the pathogenesis of HPV-negative HNSCC. *NSD1*-mutant HPV-negative HNSCC tumors represent a subset of chromatin-deregulated tumors that may be more sensitive to chemoradiotherapy, albeit more resistant to immunotherapeutic interventions compared to *NSD1*-wild-type tumors. Prospective clinical studies would be required to establish the chemoradiosensitizing effect of *NSD1* mutations in HPV-negative HNSCC. Furthermore, *NSD1*-mutant HPV-negative HNSCC patients may be sensitized to immunotherapy through treatment with EZH2 inhibitors. *NSD3*-amplified HPV-negative HNSCC tumors may be sensitive to bromodomain inhibition and possibly to cell cycle inhibitors. Further investigations into the role of NSD1, NSD2, and NSD3 in the context of HPV-negative and HPV-positive HNSCC are critical for a deeper understanding of the function of these promising therapeutic targets in the pathogenesis of HNSCC. 

## Figures and Tables

**Table 1:** Histone and non-histone substrates of the NSDs.

NSD Type	Substrate	Enzyme Activity	References
	Histone substrates		
NSD1, 2, 3	H3K36	Mono- or di-methylation of H3K36	[9,10,11,12,13]
NSD2	H1K85	Mono-methylation of H1K85	[48]
	Non-histone substrates		
NSD1	NF-κB	Methylation of K218 and K221 of the RELA/p65 subunit of NF-κB	[49]
NSD2	PTEN	Methylation of PTEN at K349	[50]
NSD3	IRF3	Methylation of IRF3 through PWWP1 domain at K366	[51]
NSD3	EGFR	Methylation of EGFR at K721	[52]

**Table 2:** Function of NSDs in HPV-negative HNSCC.

NSD Type	Function	References
NSD1	Differentiation arrest Sensitivity to cisplatin-based chemotherapy Induction of a cold immune phenotype	[57,58,59,60,61,62,63]
NSD2	Promotion of cell cycling through H3K36me2-mediating transcriptional upregulation of NEK7 Cell proliferation and survival, induction of stemness features through methylation of H1K85, and upregulation of OCT4	[48,64]
NSD3	Promotion of G1 to S phase transition through H3K36 dimethylation and upregulation of CDC6 and CDK2 Activation of ERK signaling pathway through methylation of EGFR at K721, promotion of DNA synthesis, and resistance to EGFR inhibition	[52,65]

**Table 3:** NSD inhibitors.

NSD Type	Inhibitor	Mechanism of Action	References
	Non-bromodomain inhibitors		
NSD1, 2, 3	Suramin	Nonspecific inhibitor of DNA-binding proteins	[71]
NSD2	UNC6934	Targets the PWWP1 domain of NSD2, disrupting its interaction with H3K36me2 nucleosomes	[72]
	Bromodomain inhibitors		
NSD3	(+)-JQ1	Binds to all bromodomains within the BET family	[73]
NSD3	Birabresib (OTX015)	Bromodomain inhibitor for BRD2, BRD3, and BRD4	[74]
NSD3	Pelabresib (CPI-0610)	Selective benzoisoxaoloazepine BET bromodomain inhibitor for BRD4-BD1	[75]
NSD3 NSD3	AZD 5153 6-hydroxy 2-naphthoic acid ZEN-3694	Orally available BET-BRD4 bromodomain inhibitor Orally available pan-BET bromodomain inhibitor	[76] [77]

## References

[1] Siegel R.L., Miller K.D., Jemal A. (2020). Cancer statistics, 2020. CA Cancer J. Clin..

[2] Ang K.K., Sturgis E.M. (2012). Human Papillomavirus as a Marker of the Natural History and Response to Therapy of Head and Neck Squamous Cell Carcinoma. Semin. Radiat. Oncol..

[3] Chaturvedi A.K., Engels E.A., Pfeiffer R.M., Hernandez B.Y., Xiao W., Kim E., Jiang B., Goodman M.T., Sibug-Saber M., Cozen W. (2011). Human Papillomavirus and Rising Oropharyngeal Cancer Incidence in the United States. J. Clin. Oncol..

[4] Gao J., Aksoy B.A., Dogrusoz U., Dresdner G., Gross B.E., Sumer S.O., Sun Y., Jacobsen A., Sinha R., Larsson E. (2013). Integrative Analysis of Complex Cancer Genomics and Clinical Profiles Using the cBioPortal. Sci. Signal..

[5] Cerami E., Gao J., Dogrusoz U., Gross B.E., Sumer S.O., Aksoy B.A., Jacobsen A., Byrne C.J., Heuer M.L., Larsson E. (2012). The cBio cancer genomics portal: An open platform for exploring multidimensional cancer genomics data. Cancer Discov..

[6] Leemans C.R., Snijders P.J.F., Brakenhoff R.H. (2018). The molecular landscape of head and neck cancer. Nat. Rev. Cancer.

[7] Saloura V., Vougiouklakis T., Sievers C., Burkitt K., Nakamura Y., Hager G., van Waes C. (2018). The role of protein methyltransferases as potential novel therapeutic targets in squamous cell carcinoma of the head and neck. Oral Oncol..

[8] Kim J.Y., Kee H.J., Choe N.W., Kim S.M., Eom G.H., Baek H.J., Kook H., Seo S.B. (2008). Multiple-myeloma-related WHSC1/MMSET isoform RE-IIBP is a histone methyltransferase with transcriptional repression activity. Mol. Cell. Biol..

[9] Marango J., Shimoyama M., Nishio H., Meyer J.A., Min D.-J., Sirulnik A., Martinez-Martinez Y., Chesi M., Bergsagel P.L., Zhou M.-M. (2008). The MMSET protein is a histone methyltransferase with characteristics of a transcriptional corepressor. Blood.

[10] Li Y., Trojer P., Xu C.-F., Cheung P., Kuo A., Drury W.J., Qiao Q., Neubert T.A., Xu R.-M., Gozani O. (2009). The Target of the NSD Family of Histone Lysine Methyltransferases Depends on the Nature of the Substrate. J. Biol. Chem..

[11] Nimura K., Ura K., Shiratori H., Ikawa M., Okabe M., Schwartz R.J., Kaneda Y. (2009). A histone H3 lysine 36 trimethyltransferase links Nkx2-5 to Wolf–Hirschhorn syndrome. Nature.

[12] Lucio-Eterovic A.K., Singh M.M., Gardner J.E., Veerappan C.S., Rice J.C., Carpenter P.B. (2010). Role for the nuclear receptor-binding SET domain protein 1 (NSD1) methyltransferase in coordinating lysine 36 methylation at histone 3 with RNA polymerase II function. Proc. Natl. Acad. Sci. USA.

[13] Kuo A.J., Cheung P., Chen K., Zee B.M., Kioi M., Lauring J., Xi Y., Park B.H., Shi X., Garcia B.A. (2011). NSD2 links demethylation of histone H3 at lysine 36 to oncogenic programming. Mol. Cell.

[14] Qiao Q., Li Y., Chen Z., Wang M., Reinberg D., Xu R.M. (2011). The structure of NSD1 reveals an autoregulatory mechanism underlying histone H3K36 methylation. J. Biol. Chem..

[15] Rahman S., Sowa M.E., Ottinger M., Smith J.A., Shi Y., Harper J.W., Howley P.M. (2011). The Brd4 Extraterminal Domain Confers Transcription Activation Independent of pTEFb by Recruiting Multiple Proteins, Including NSD3. Mol. Cell. Biol..

[16] Seiwert T.Y., Zuo Z., Keck M.K., Khattri A., Pedamallu C.S., Stricker T., Brown C., Pugh T.J., Stojanov P., Cho J. (2015). Integrative and Comparative Genomic Analysis of HPV-Positive and HPV-Negative Head and Neck Squamous Cell Carcinomas. Clin. Cancer Res..

[17] Bennett R., Swaroop A., Troche C., Licht J.D. (2017). The Role of Nuclear Receptor–Binding SET Domain Family Histone Lysine Methyltransferases in Cancer. Cold Spring Harb. Perspect. Med..

[18] Vougiouklakis T., Hamamoto R., Nakamura Y., Saloura V. (2015). The NSD family of protein methyltransferases in human cancer. Epigenomics.

[19] Jaju R.J., Fidler C., Haas O.A., Strickson A.J., Watkins F., Clark K., Cross N.C., Cheng J.F., Aplan P.D., Kearney L. (2001). A novel gene, NSD1, is fused to NUP98 in the t(5;11)(q35;p15.5) in de novo childhood acute myeloid leukemia. Blood.

[20] Shiba N., Ichikawa H., Taki T., Park M.J., Jo A., Mitani S., Kobayashi T., Shimada A., Sotomatsu M., Arakawa H. (2013). NUP98-NSD1 gene fusion and its related gene expression signature are strongly associated with a poor prognosis in pediatric acute myeloid leukemia. Genes Chromosomes Cancer.

[21] Wang G.G., Cai L., Pasillas M.P., Kamps M.P. (2007). NUP98-NSD1 links H3K36 methylation to Hox-A gene activation and leukaemogenesis. Nat. Cell Biol..

[22] Stec I., Wright T.J., van Ommen G.J.B., de Boer P.A., van Haeringen A., Moorman A.F., Altherr M.R., den Dunnen J.T. (1998). WHSC1, a 90 kb SET domain-containing gene, expressed in early development and homologous to a Drosophila dysmorphy gene maps in the Wolf–Hirschhorn syndrome critical region and is fused to IgH in t(4;14) multiple myeloma. Hum. Mol. Genet..

[23] Chesi M., Nardini E., Lim R.S., Smith K.D., Kuehl W.M., Bergsagel P.L. (1998). The t(4;14) translocation in myeloma dysregulates both FGFR3 and a novel gene, MMSET, resulting in IgH/MMSET hybrid transcripts. Blood.

[24] Maiolo A.T., Neri A., Finelli P., Fabris S., Zagano S., Baldini L., Intini D., Nobili L., Lombardi L. (1999). Detection of t(4;14)(p16.3;q32) chromosomal translocation in multiple myeloma by double-color fluorescent in situ hybridization. Blood.

[25] Lauring J., Abukhdeir A.M., Konishi H., Garay J.P., Gustin J.P., Wang Q., Arceci R.J., Matsui W., Park B.H. (2008). The multiple myeloma–associated MMSET gene contributes to cellular adhesion, clonogenic growth, and tumorigenicity. Blood.

[26] Brito J.L., Walker B., Jenner M., Dickens N.J., Brown N.J., Ross F.M., Avramidou A., Irving J.A., Gonzalez D., Davies F.E. (2009). MMSET deregulation affects cell cycle progression and adhesion regulons in t(4;14) myeloma plasma cells. Haematologica.

[27] Martinez-Garcia E., Popovic R., Min D.-J., Sweet S., Thomas P., Zamdborg L., Heffner A., Will C., Lamy L., Staudt L.M. (2011). The MMSET histone methyl transferase switches global histone methylation and alters gene expression in t(4;14) multiple myeloma cells. Blood.

[28] Huang Z., Wu H., Chuai S., Xu F., Yan F., Englund N., Wang Z., Zhang H., Fang M., Wang Y. (2013). NSD2 Is Recruited through Its PHD Domain to Oncogenic Gene Loci to Drive Multiple Myeloma. Cancer Res..

[29] Shah M.Y., Martinez-Garcia E., Phillip J.M., Chambliss A.B., Popovic R., Ezponda T., Small E.C., Will C., Phillip M.P., Neri P. (2016). MMSET/WHSC1 enhances DNA damage repair leading to an increase in resistance to chemotherapeutic agents. Oncogene.

[30] Zheng Y., Sweet S.M.M., Popovic R., Martinez-Garcia E., Tipton J.D., Thomas P.M., Licht J.D., Kelleher N.L. (2012). Total kinetic analysis reveals how combinatorial methylation patterns are established on lysines 27 and 36 of histone H3. Proc. Natl. Acad. Sci. USA.

[31] Angrand P.-O., Apiou F., Stewart A., Dutrillaux B., Losson R., Chambon P. (2001). NSD3, a New SET Domain-Containing Gene, Maps to 8p12 and Is Amplified in Human Breast Cancer Cell Lines. Genomics.

[32] Stec I., van Ommen G.-J.B., den Dunnen J.T. (2001). WHSC1L1, on Human Chromosome 8p11.2, Closely Resembles WHSC1 and Maps to a Duplicated Region Shared with 4p16.3. Genomics.

[33] Chen Y., McGee J., Chen X., Doman T.N., Gong X., Zhang Y., Hamm N., Ma X., Higgs R.E., Bhagwat S.V. (2014). Identification of Druggable Cancer Driver Genes Amplified across TCGA Datasets. PLoS ONE.

[34] Yang Z.-Q., Liu G., Bollig-Fischer A., Giroux C.N., Ethier S.P. (2010). Transforming Properties of 8p11-12 Amplified Genes in Human Breast Cancer. Cancer Res..

[35] Zhou Z., Thomsen R., Kahns S., Nielsen A.L. (2010). The NSD3L histone methyltransferase regulates cell cycle and cell invasion in breast cancer cells. Biochem. Biophys. Res. Commun..

[36] Kang D., Cho H.S., Toyokawa G., Kogure M., Yamane Y., Iwai Y., Hayami S., Tsunoda T., Field H.I., Matsuda K. (2013). The histone methyltransferase Wolf–Hirschhorn syndrome candidate 1-like 1 (WHSC1L1) is involved in human carcinogenesis. Genes Chromosomes Cancer.

[37] Yuan G., Flores N.M., Hausmann S., Lofgren S.M., Kharchenko V., Angulo-Ibanez M., Sengupta D., Lu X., Czaban I., Azhibek D. (2021). Elevated NSD3 histone methylation activity drives squamous cell lung cancer. Nature.

[38] Baker L.A., Allis C.D., Wang G.G. (2008). PHD fingers in human diseases: Disorders arising from misinterpreting epigenetic marks. Mutat. Res. Mol. Mech. Mutagen..

[39] Pasillas M.P., Shah M., Kamps M.P. (2010). NSD1 PHD domains bind methylated H3K4 and H3K9 using interactions disrupted by point mutations in human sotos syndrome. Hum. Mutat..

[40] Sankaran S.M., Wilkinson A.W., Elias J.E., Gozani O. (2016). A PWWP Domain of Histone-Lysine N-Methyltransferase NSD2 Binds to Dimethylated Lys-36 of Histone H3 and Regulates NSD2 Function at Chromatin. J. Biol. Chem..

[41] Morishita M., di Luccio E. (2011). Cancers and the NSD family of histone lysine methyltransferases. Biochim. Biophys. Acta.

[42] Dillon S.C., Zhang X., Trievel R.C., Cheng X. (2005). The SET-domain protein superfamily: Protein lysine methyltransferases. Genome Biol..

[43] Herz H.-M., Garruss A., Shilatifard A. (2013). SET for life: Biochemical activities and biological functions of SET domain-containing proteins. Trends Biochem. Sci..

[44] Huang N., Baur E.V., Garnier J., Lerouge T., Vonesch J., Lutz Y., Chambon P., Losson R. (1998). Two distinct nuclear receptor interaction domains in NSD1, a novel SET protein that exhibits characteristics of both corepressors and coactivators. EMBO J..

[45] Rao B., Shibata Y., Strahl B.D., Lieb J.D. (2005). Dimethylation of Histone H3 at Lysine 36 DemarcatesRegulatory and Nonregulatory ChromatinGenome-Wide. Mol. Cell. Biol..

[46] Wagner E.J., Carpenter P.B. (2012). Understanding the language of Lys36 methylation at histone H3. Nat. Rev. Mol. Cell Biol..

[47] Li W., Tian W., Yuan G., Deng P., Sengupta D., Cheng Z., Cao Y., Ren J., Qin Y., Zhou Y. (2020). Molecular basis of nucleosomal H3K36 methylation by NSD methyltransferases. Nature.

[48] Saloura V., Vougiouklakis T., Bao R., Kim S., Baek S., Zewde M., Bernard B., Burkitt K., Nigam N., Izumchenko E. (2020). WHSC1 monomethylates histone H1 and induces stem-cell like features in squamous cell carcinoma of the head and neck. Neoplasia.

[49] Lu T., Jackson M.W., Wang B., Yang M., Chance M.R., Miyagi M., Gudkov A.V., Stark G.R. (2009). Regulation of NF-κB by NSD1/FBXL11-dependent reversible lysine methylation of p65. Proc. Natl. Acad. Sci. USA.

[50] Zhang J., Lee Y.-R., Dang F., Gan W., Menon A.V., Katon J.M., Hsu C.-H., Asara J.M., Tibarewal P., Leslie N.R. (2019). PTEN Methylation by NSD2 Controls Cellular Sensitivity to DNA Damage. Cancer Discov..

[51] Wang C., Wang Q., Xu X., Xie B., Zhao Y., Li N., Cao X. (2017). The methyltransferase NSD3 promotes antiviral innate immunity via direct lysine methylation of IRF3. J. Exp. Med..

[52] Saloura V., Vougiouklakis T., Zewde M., Deng X., Kiyotani K., Park J.-H., Matsuo Y., Lingen M., Suzuki T., Dohmae N. (2017). WHSC1L1-mediated EGFR mono-methylation enhances the cytoplasmic and nuclear oncogenic activity of EGFR in head and neck cancer. Sci. Rep..

[53] Rayasam G.V., Wendling O., Angrand P.-O., Mark M., Niederreither K., Song L., Lerouge T., Hager G.L., Chambon P., Losson R. (2003). NSD1 is essential for early post-implantation development and has a catalytically active SET domain. EMBO J..

[54] Kurotaki N., Imaizumi K., Harada N., Masuno M., Kondoh T., Nagai T., Ohashi H., Naritomi K., Tsukahara M., Makita Y. (2002). Haploinsufficiency of NSD1 causes Sotos syndrome. Nat. Genet..

[55] Leventopoulos G., Kitsiou-Tzeli S., Kritikos K., Psoni S., Mavrou A., Kanavakis E., Fryssira H. (2009). A Clinical Study of Sotos Syndrome Patients With Review of the Literature. Pediatr. Neurol..

[56] Rauch A., Schellmoser S., Kraus C., Dorr H.G., Trautmann U., Altherr M.R., Pfeiffer R.A., Reis A. (2001). First known microdeletion within the Wolf-Hirschhorn syndrome critical region refines genotype-phenotype correlation. Am. J. Med. Genet..

[57] Papillon-Cavanagh S., Lu C., Gayden T., Mikael L.G., Bechet D., Karamboulas C., Ailles L., Karamchandani J., Marchione D., Garcia B.A. (2017). Impaired H3K36 methylation defines a subset of head and neck squamous cell carcinomas. Nat. Genet..

[58] Pan C., Izreig S., Yarbrough W.G., Issaeva N. (2019). NSD1 mutations by HPV status in head and neck cancer: Differences in survival and response to DNA-damaging agents. Cancers Head Neck.

[59] Peri S., Izumchenko E., Schubert A.D., Slifker M.J., Ruth K., Serebriiskii I.G., Guo T., Burtness B.A., Mehra R., Ross E.A. (2017). NSD1- and NSD2-damaging mutations define a subset of laryngeal tumors with favorable prognosis. Nat. Commun..

[60] Bui N., Huang J.K., Bojorquez-Gomez A., Licon K., Sanchez K.S., Tang S.N., Beckett A.N., Wang T., Zhang W., Shen J.P. (2018). Disruption of *NSD1* in Head and Neck Cancer Promotes Favorable Chemotherapeutic Responses Linked to Hypomethylation. Mol. Cancer Ther..

[61] Farhangdoost N., Horth C., Hu B., Bareke E., Chen X., Li Y., Coradin M., Garcia B.A., Lu C., Majewski J. (2021). Chromatin dysregulation associated with NSD1 mutation in head and neck squamous cell carcinoma. Cell Rep..

[62] Brennan K., Shin J.H., Tay J.K., Prunello M., Gentles A.J., Sunwoo J.B., Gevaert O. (2017). NSD1 inactivation defines an immune cold, DNA hypomethylated subtype in squamous cell carcinoma. Sci. Rep..

[63] Li Y., Goldberg E.M., Chen X., Xu X., McGuire J.T., Leuzzi G., Karagiannis D., Tate T., Farhangdoost N., Horth C. (2022). Histone methylation antagonism drives tumor immune evasion in squamous cell carcinomas. Mol. Cell.

[64] Saloura V., Cho H.-S., Kiyotani K., Alachkar H., Zuo Z., Nakakido M., Tsunoda T., Seiwert T., Lingen M., Licht J. (2015). WHSC1 Promotes Oncogenesis through Regulation of NIMA-Related Kinase-7 in Squamous Cell Carcinoma of the Head and Neck. Mol. Cancer Res..

[65] Saloura V., Vougiouklakis T., Zewde M., Kiyotani K., Park J.-H., Gao G., Karrison T., Lingen M., Nakamura Y., Hamamoto R. (2016). WHSC1L1 drives cell cycle progression through transcriptional regulation of CDC6 and CDK2 in squamous cell carcinoma of the head and neck. Oncotarget.

[66] Xue W., Shen Z., Li L., Zheng Y., Yan D., Kan Q., Zhao J. (2020). Long non-coding RNAs MACC1-AS1 and FOXD2-AS1 mediate NSD2-induced cisplatin resistance in esophageal squamous cell carcinoma. Mol. Ther. Nucleic Acids.

[67] French C.A. (2018). NUT Carcinoma: Clinicopathologic features, pathogenesis, and treatment. Pathol. Int..

[68] French C.A., Rahman S., Walsh E.M., Kühnle S., Grayson A.R., Lemieux M.E., Grunfeld N., Rubin B.P., Antonescu C.R., Zhang S. (2014). NSD3–NUT Fusion Oncoprotein in NUT Midline Carcinoma: Implications for a Novel Oncogenic Mechanism. Cancer Discov..

[69] Haft S., Ren S., Xu G., Mark A., Fisch K., Guo T.W., Khan Z., Pang J., Ando M., Liu C. (2019). Mutation of chromatin regulators and focal hotspot alterations characterize human papillomavirus–positive oropharyngeal squamous cell carcinoma. Cancer.

[70] Gameiro S.F., Ghasemi F., Zeng P.Y.F., Mundi N., Howlett C.J., Plantinga P., Barrett J.W., Nichols A.C., Mymryk J.S. (2021). Low expression of NSD1, NSD2, and NSD3 define a subset of human papillomavirus-positive oral squamous carcinomas with unfavorable prognosis. Infect. Agents Cancer.

[71] Allali-Hassani A., Kuznetsova E., Hajian T., Wu H., Dombrovski L., Li Y., Gräslund S., Arrowsmith C.H., Schapira M., Vedadi M. (2014). A Basic Post-SET Extension of NSDs Is Essential for Nucleosome Binding In Vitro. SLAS Discov. Adv. Sci. Drug Discov..

[72] Dilworth D., Hanley R.P., de Freitas R.F., Allali-Hassani A., Zhou M., Mehta N., Marunde M.R., Ackloo S., Machado R.A.C., Yazdi A.K. (2021). A chemical probe targeting the PWWP domain alters NSD2 nucleolar localization. Nat. Chem. Biol..

[73] Filippakopoulos P., Qi J., Picaud S., Shen Y., Smith W.B., Fedorov O., Morse E.M., Keates T., Hickman T.T., Felletar I. (2010). Selective inhibition of BET bromodomains. Nature.

[74] Noel J.K., Iwata K., Ooike S., Sugahara K., Nakamura H., Daibata M. (2013). Development of the BET bromodomain inhibitor OTX015. Mol. Cancer Ther..

[75] Albrecht B.K., Gehling V.S., Hewitt M.C., Vaswani R.G., Côté A., Leblanc Y., Nasveschuk C.G., Bellon S., Bergeron L., Campbell R. (2016). Identification of a Benzoisoxazoloazepine Inhibitor (CPI-0610) of the Bromodomain and Extra-Terminal (BET) Family as a Candidate for Human Clinical Trials. J. Med. Chem..

[76] Rhyasen G.W., Hattersley M.M., Yao Y., Dulak A., Wang W., Petteruti P., Dale I.L., Boiko S., Cheung T., Zhang J. (2016). AZD5153: A Novel Bivalent BET Bromodomain Inhibitor Highly Active against Hematologic Malignancies. Mol. Cancer Ther..

[77] Aggarwal R.R., Schweizer M.T., Nanus D.M., Pantuck A.J., Heath E.I., Campeau E., Attwell S., Norek K., Snyder M., Bauman L. (2020). A Phase Ib/IIa Study of the Pan-BET Inhibitor ZEN-3694 in Combination with Enzalutamide in Patients with Metastatic Castration-resistant Prostate Cancer. Clin. Cancer Res..

